# Optimizing predictive maintenance and mission assignment to enhance fleet readiness under uncertainty

**DOI:** 10.1007/s43684-025-00104-1

**Published:** 2025-08-15

**Authors:** Ryan O’Neil, Abdelhakim Khatab, Claver Diallo

**Affiliations:** 1 Department of Industrial Engineering, Dalhousie University, 5269 Morris st. PO Box 15000, Halifax, Nova Scotia B3H-4R2 Canada; 2 Lorraine University-LGIPM, 3 rue Augustin Fresnel BP 45112, 57073 Metz Cedex 03, France

**Keywords:** Fleet Selective Maintenance, Stochastic Optimization, Predictive Maintenance, Data-driven Prognostics, Reliability Modeling

## Abstract

In many industrial settings, fleets of assets are required to operate through alternating missions and breaks. Fleet Selective Maintenance (FSM) is widely used in such contexts to improve the fleet performance. However, existing FSM models assume that upcoming missions are identical and require only a single system configuration for completion. Additionally, these models typically assume that all missions must be completed, overlooking resource constraints that may prevent readying all systems within the available break duration. This makes mission prioritization and assignment a necessary consideration for the decision-maker. This work proposes a novel FSM model that jointly optimizes system to mission assignment, component and maintenance level selection, and repair task allocation. The proposed framework integrates analytical models for standard components and Deep Neural Networks (DNNs) for sensor-monitored ones, enabling a hybrid reliability assessment approach that better reflects real-world multi-component systems. To account for uncertainties in maintenance and break durations, a chance-constrained optimization model is developed to ensure that maintenance is completed within the available break duration with a specified confidence level. The optimization model is reformulated using two well-known techniques: Sample Average Approximation (SAA) and Conditional Value-at-Risk (CVaR) approximation. A case study of military aircraft fleet maintenance is investigated to demonstrate the accuracy and added value of the proposed approach.

## Introduction

Many engineered assets and systems are designed to operate according to a sequence of alternating missions and breaks. These mission-oriented systems (MOS) are ubiquitous in several industrial applications such as air transportation, military equipment, nuclear plants, spacecraft, and unmanned autonomous vehicles. A key metric used to assess their performance is the mission success probability, defined as the system’s ability to successfully complete a defined mission of a specific profile under given operational conditions. To ensure that the mission success probability meets a required level for the upcoming mission, maintenance actions are usually carried out on the system/components during scheduled maintenance breaks. However, due to the limited maintenance resources (*e.g.,* time, budget, materials, spare parts, crews) only a subset of the desired maintenance tasks can be performed. In the literature, this resource-constrained maintenance problem is known as the selective maintenance problem (SMP). The primary objective is to identify the optimal subset of components and maintenance actions that either minimize maintenance costs while ensuring a required level of the system reliability to operate the next mission, or maximize the system reliability subjected to maintenance budget and/or break duration constraints.

The original selective maintenance (SM) model was introduced in [1] with the aim of maximizing mission success probability using a full enumeration algorithm. Many researchers have since expanded upon this original work to include complex system configurations [2], multistate systems [3, 4], component dependence [5–7], uncertainties related to maintenance, break, and mission durations [8–10], uncertainties related to maintenance quality [11, 12], fleet level selective maintenance [13, 14], and multiple repair channels [15, 16]. Significant advances have also been made in the solution methods used to solve the SMP. [17] refined to the total enumeration approach developed in [1]. Multiple authors have utilized metaheuristics such as Tabu search [18], genetic algorithms [16], and ant colony optimization [9]. [2] introduced a two-phase approach that eliminates the need for solving a nonlinear objective function as the approach converts the problem to a multidimensional multi-choice knapsack problem. More recently, [19] introduced a column generation approach to solve the multi-mission SMP, where a metaheuristic was employed to solve the subproblems. [20] developed a branch-and-price framework to solve large-scale single-mission SMP problems where the subproblems were solved exactly by reformulating the problem using exponential cones.

Many existing SMP papers treat maintenance and break durations as deterministic parameters that are known and constant. Nevertheless, there are many applications in which the precise duration of the maintenance actions and break durations cannot be exactly determined but would be characterized by a random value [8, 21]. For instance, in military aircraft fleets, the unexpected start of the next mission/battle introduces uncertainties in the available maintenance time [9]. Due to the variability in repair crew experience, among many other factors, the time to perform maintenance actions may also be nondeterministic. [8] studied the SMP when durations of the maintenance actions, scheduled breaks, and missions are stochastic by proposing a nonlinear stochastic optimization problem with a chance constraint to ensure the probability of completing the maintenance actions is greater than some predefined service level. [9] studied the SMP with stochastic maintenance and mission durations for multi-state systems. Their model aims to identify the subset of maintenance actions to perform, as well as the sequencing of the actions such that the system reliability is maximized. The saddlepoint approximation is utilized to approximate the multi-dimensional convolution that arises when evaluating the system reliability [22]. [23] dealt with a multi-mission SMP for multicomponent systems where the durations of missions, maintenance actions, and breaks are stochastic. [24] investigated the SMP with stochastic duration of maintenance tasks and breaks. The authors used the expected cumulative performance to calculate system reliability. More recently, [25] studied the SMP under uncertain maintenance durations, and employed distributionally robust chance-constraints to handle such uncertainties.

An interesting and important extension of the original SMP that has received much attention is the Fleet Selective Maintenance Problem (FSMP). In the FSMP, maintenance decisions must be determined for each component within a fleet of systems. [26] first introduced the FSMP and studied its application to a fleet of military aircraft. The authors proposed an optimization model aimed at maximizing fleet reliability, which is defined as the probability that all systems successfully complete the upcoming mission. [27] extended the work of [26] by proposing a cost-based formulation and considering the possibility of canceling upcoming missions. [28] introduced a multi-objective FSMP with three unique objective functions: maximize fleet reliability, minimize total maintenance cost, and minimize gap time. The proposed model was applied to a truck fleet carrying out long-distance highway transportation missions. [13] and [29] extended the FSMP problem to include repairperson assignment decisions, and [30] proposed a resource-constrained fleet maintenance optimization model with the objective of maximizing the expected fleet readiness under stochastic break durations.

Despite the above-mentioned extensions, several key limitations remain in the existing FSMP literature. First, all FSMP models rely on a restrictive assumption according to which missions are identical and require a single system configuration for successful completion. However, this assumption does not hold in many real-world scenarios. For example, in a military aircraft fleet, missions often require different system configurations and have different durations in accordance with mission goals [31]. Nearly all proposed models assume that all upcoming missions must be completed. However, due to resource limitations, it may not be possible to ready all systems within the available break duration, making mission prioritization and assignment a necessary consideration. Additionally, all FSMP models mentioned above fail to consider the inherent uncertainties in both maintenance and break durations. Finally, the FSMP models that have been discussed thus far rely on statistical/model-driven methods for system reliability computation. However, with the recent advances in data collection with sensors and the advent of Industrial Internet of Things (IIoT), data-driven methods relying on deep neural networks have become increasingly popular for the remaining useful life (RUL) prediction and reliability assessment [32]. These methods have achieved state-of-the-art performance in terms of prognostics and predictive maintenance. Although data-driven selective maintenance strategies have recently been studied, the few existing works [33, 34] assumed that all system components are sensor monitored. In practice, however, many industrial systems are composed of both standard components with known lifetime distributions, and critical components which are sensor-monitored. Thus, a hybrid approach is needed for the reliability modeling and assessment of such systems. These limitations motivate the need for the development of a generalized FSMP framework that incorporates stochastic maintenance and break durations, mission prioritization and assignment, and hybrid reliability assessment techniques.

Aiming to help maintenance decision-makers resolve practical issues arising in the implementation of the FSMP, this paper develops a new approach where the classical FSMP is extended to include flexible system configuration adapted to multiple mission types with unique operational demands, reliability requirements, minimum system assignment thresholds, and associated penalties. Maintenance activities are permitted during the break duration to ensure that systems assigned to specific missions meet the pre-specified performance requirements. Unlike existing studies, our approach introduces a new FSMP model that jointly determines four key decisions: (1) the assignment of systems to mission types, (2) the selection of components within each system to maintain, (3) the corresponding maintenance levels to be performed on the selected components, and (4) the assignment of maintenance tasks to repairpersons. Furthermore, inevitable uncertainties in maintenance and break durations are also explicitly accounted for. A chance-constrained optimization model is then developed and reformulated. A fleet of identical multi-component systems is considered, where components are of two types: standard and critical. The first type of components (*i.e.,* standard) are assumed to follow known lifetime distributions allowing for the analytical reliability calculations. The second type of components (*i.e.,* critical) are sensor-monitored with reliability estimates obtained using a Deep Neural Network (DNN) with Monte Carlo Dropout (MCD), a technique that has demonstrated outstanding performance in predictive maintenance [33, 35, 36].

A preliminary version of the present work appeared in a conference paper [22] where the SMP is solved for a single MOS operating under uncertain maintenance and break durations. The resulting optimization model maximized the system reliability for the next mission. The present paper is a significant extension of [22] as it expands the complexity of the model and analyses. Major contributions of the extended paper include: i) an expanded literature review covering recent advances in SMP; ii) an extension from a single MOS to a fleet of several MOS, where systems are operating under break duration uncertainties; iii) systems are composed of two types of components and Deep Neural Networks (DNNs) are used to predict the RUL of sensor-monitored components; iv) a new FSMP formulation and efficient solution method are presented; v) several numerical experiments are conducted to highlight the proposed approach.

In summary, this paper advances the FSMP literature through the following contributions and novel modeling and methodological elements:

- The development of an extended FSMP framework that incorporates flexible system configuration across multiple mission types, each characterized by a required reliability threshold, systems’ configuration and assignment, and associated penalties.
- The consideration of two types of components, standard and critical components. A data-driven reliability estimation method is developed using DNN combined with MCD for critical components.
- The development of a novel joint optimization model that simultaneously determines (i) the assignment of systems to mission types, (ii) the selection of components to maintain, (iii) the maintenance levels to perform, and (iv) the allocation of maintenance tasks to available repairpersons.
- The consideration of uncertainties in both maintenance tasks and break durations. A chance-constrained optimization model is developed, and then reformulated using Sample Average Approximation (SAA) and the Conditional Value-at-Risk (CVaR) approximation.

This paper is organized as follows. Section 2 provides a comprehensive description of the system and the problem under investigation. This section also develops the reliability and maintenance models for both standard and sensor-monitored components. In Sect. 3, the novel deterministic formulation of the FSMP is introduced, while Sect. 4 presents the chance-constrained formulation and its CVaR approximation. The mathematical model is validated through a case study involving the maintenance of a fleet of military aircraft in Sect. 5. The numerical results are analyzed and extensively discussed in Sect. 6. Finally, Sect. 7 concludes the paper by summarizing the key findings and identifying promising directions for future research.

## Problem description

In this section, the problem considered is described and the main working assumptions are presented. First, the notation system is introduced.

### Notation

The following notation system is used in the modeling of the problem.

*Sets:*

$\mathcal{I}$Set of systems, $\mathcal{I} = \{1, \ldots , I\}$, with index *i*.

$\mathcal{J}$Set of subsystems per system. $\mathcal{J} = \{1, \ldots , J\}$, with index *j*.

$\mathcal{J}_{m}$Set of subsystems required to perform mission type *m*. $\mathcal{J}_{m} \subseteq \mathcal{J}$.

$\mathcal{K}_{j}$Set of all components in subsystem *j*. $\mathcal{K}_{j} = \{1, \ldots , J_{j}\}$, with index *k*.

$\mathcal{K}^{\text{s}}_{j}$Set of standard components in subsystem *j*. $\mathcal{K}^{\text{s}}_{j} \subseteq \mathcal{K}_{j}$, with index *k*.

$\bar{\mathcal{K}}^{\text{s}}_{j}$Set of sensor-monitored components in subsystem *j*. $\bar{\mathcal{K}}^{s}_{j}=\mathcal{K}_{j} \setminus \mathcal{K}_{j}^{s}$.

$\mathcal{L}_{ijk}$Set of maintenance levels available for component $E_{ijk}$; $\mathcal{L}_{ijk} = \{1, \ldots , L_{ijk}\}$, with index *l*.

$\mathcal{Q}$Set of repairpersons, $\mathcal{Q} = \{1, \ldots , Q\}$, with index *q*.

$\mathcal{M}$Set of mission types, $\mathcal{M} = \{1, \ldots , M\}$, with index *m*.

*Parameters:*

*I*Number of systems within the fleet.

*J*Number of subsystems per system.

$J_{j}$Number of components in subsystem *j*.

$E_{ijk}$The $k\textsuperscript{th}$ component in subsystem *j* of system *i*.

$B_{ijk}$Age of component $E_{ijk}$ at the start of the break.

$u_{ijk}$Status of $E_{ijk}$ at the start of the break.

$L_{ijk}$Number of maintenance levels available for component $E_{ijk}$.

*D*Duration of maintenance break.

*Q*Maximum number of repair persons available.

$c^{f}$Fixed cost of hiring/using a repairperson.

$c^{v}$Variable cost rate of using a repairperson.

$t^{p}_{ijkl}$Time to carry out PM level *l* on component $E_{ijk}$.

$t^{c}_{ijkl}$Time to carry out CM level *l* on component $E_{ijk}$.

*M*Number of mission types.

$U_{m}$Length of mission type *m*.

$\rho _{m}$Penalty incurred for not completing mission type *m*.

${R}^{\text{min}}_{jm}$Required minimum reliability for subsystem *j* for mission type *m*.

*Decision variables:*

$\chi _{m}$Binary variable equal to 1 if mission type *m* is completed.

$\gamma _{q}$Binary variable equal to 1 if repair person *q* is utilized.

$z_{im}$Binary variable equal to 1 if system *i* is assigned to mission type *m*.

$x_{ijklq}$Binary variable equal to 1 if repairperson *q* performs maintenance level *l* on component $E_{ijk}$.

### Main working assumptions

1. Components and systems are binary state, this assumption is appropriate for a wide range of systems and is commonly used in the literature [14, 16, 37].
2. During the maintenance break the systems are assumed to be switched off and therefore not experiencing any degradation. This is reasonable and is commonly used [15, 33].
3. Maintenance activities are allowed only during the break duration. This is consistent with the definition of the SMP [2, 26, 37].
4. Maintenance and break durations are stochastic quantities with known probability distributions. This is reasonable and commonly used [9, 10, 38].

### Problem description

The FSMP addressed in this work deals with the optimization of the selective maintenance planning, repairperson scheduling, as well as system to mission assignment. Although this problem is inspired by the maintenance scheduling challenges encountered in maintaining military aircraft fleets, it can easily be adapted to various heterogeneous fleet of assets performing mission-critical operations. Examples could include: i) a fleet of various size autonomous mobile robots [39] equipped with mission-specific subsystems or; ii) a heterogeneous fleet of delivery trucks running short or long routes with varying loading/unloading equipment requirements during the day and undergoing maintenance at night, and; iii) a fleet of military aircraft that undergo maintenance between reconnaissance or humanitarian support missions requiring different equipment performance.

Specifically, it is assumed that a fleet of systems has just entered a maintenance break, providing the opportunity to perform necessary maintenance actions to get the fleet ready for a set of various upcoming missions. During this break, the goal is to determine the assignment of systems to mission types, the set of components within each system that should be maintained, and the required number of repairpersons needed to carry out the selected maintenance plan, the assignment of maintenance tasks to repairpersons. The objective is to minimize the grand total cost, which includes penalties for unselected missions as well as fixed and variable costs related to selected maintenance actions. Maintenance plays an important role in ensuring systems are mission-ready by addressing reliability requirements. A system can only be assigned to a mission provided that its reliability meets the required threshold. Each mission type has unique operational demands, reliability requirements, minimum system assignment thresholds, and associated penalties. However, due to resource limitations, such as the availability of repairpersons and time constraints, only a subset of all desirable maintenance actions can be performed. In the FSMP addressed, maintenance and break durations are stochastic, following known probability distributions. The uncertainty in maintenance task and break durations impacts planning and assignment decisions by introducing the risk of potentially not completing the required maintenance actions during the break and thus delaying mission readiness. Effective maintenance planning and the overall assignment decisions must indeed account for this uncertainty to ensure cost-effective and robust solutions.

Each mission type $m \in \mathcal{M}$ is characterized by a profile captured by the vector $(\rho _{m}, U_{m}, \zeta _{m}, \mathcal{J}_{m}, \mathcal{R}_{m})$, where $\rho _{m}$ is the penalty incurred for not completing mission *m*, $U_{m}$ is the mission duration, and $\zeta _{m}$ is the minimum number of systems required to successfully operate mission *m*. The parameter set $\mathcal{J}_{m}$ corresponds to the subset of subsystems or functions that are required to perform mission *m*. For example, in the context of a military aircraft fleet, a logistics support mission would likely not require the functionality of weapon systems, whereas a combat mission certainly would. Consequently, a system assigned to a logistics support mission would not require maintenance on those subsystems. Finally, the parameter $\mathcal{R}_{m}$ defines the set of minimum reliability requirements for all subsystems in the subset $\mathcal{J}_{m}$. To perform the different mission types $m \in \mathcal{M}$, a fleet of *I* identical multi-component MOS is available. Each MOS *i* is comprised of *J* subsystems in series, and each subsystem is composed of $J_{j}$ statistically independent components $E_{ijk}$ in parallel (*i.e.*, subsystem *j* functions if at least 1 out of its $J_{j}$ components is functioning). At the start of the current maintenance break, each component $E_{ijk}$ is characterized by its current effective age $B_{ijk}$, while its status is determined by the binary state variable $u_{ijk}$ defined as follows:

1 $$ {u_{ijk}}= \textstyle\begin{cases} 1, & \text{if }E_{ijk}\text{ is functioning at the start of the} \\ &\text{break}, \\ 0, & \text{otherwise.} \end{cases} $$

The components within each system are divided into two sets based on the availability of reliability information: standard components, defined by the set $\mathcal{K}^{\text{s}}_{j}$, have lifetime distributions following known probability distributions allowing their reliability to be computed analytically. Sensor-monitored components, defined by the set $\bar{\mathcal{K}}^{\text{s}}_{j}$ are equipped with sensors that provide real-time condition monitoring data (*e.g.,* vibration, temperature, wear). These data are used to predict the RUL of each component. Data-driven approaches relying on deep neural networks (DNNs) have shown exceptional performance in predicting the probability of failure of such components [32]. In the present work, a DNN with MCD is used to estimate the probability that these sensor-monitored components will successfully operate the assigned mission. The hybrid (dual) model developed here addresses a real practical consideration: it is uneconomical and unpractical to have sensors on every single component of a multicomponent system. Some important/critical components will be equipped with sensors for real time condition monitoring, while the reliability of standard (non-critical) ones will simply be characterized/estimated using historical field data via lifetime probability distributions. The proposed dual/hybrid methodology is more general and can reduce to the two extreme cases: i) all components have sensors (pure predictive maintenance or data-driven) or ii) no component has sensor (pure lifetimes probability distributions based reliability assessment or analytical). A detailed description of the reliability computation for both standard and sensor-monitored components is provided in the next subsection.

### Reliability computation

System *i* can only be assigned to mission *m* if and only if each subsystem *j* meets the required subsystem reliability threshold ${R}^{\text{min}}_{jm}$ for that mission type. For each mission *m*, specific reliability requirements $\mathcal{R}_{m}$ exist for different functions or subsystems $\mathcal{J}_{m}$. To compute the reliability of each subsystem $j\in \mathcal{J}_{m}$, the reliability of each component $E_{ijk}$ must first be determined. If $E_{ijk}$ is a standard component with known lifetime distributions and effective age $B_{ijk}$, then its reliability $\mathcal{R}_{ijk}(t|{B_{ijk}})$ is computed as:

2 $$ \mathcal{R}_{ijk}(t|{B_{ijk}})= \frac{\mathcal{R}_{ijk}({B_{ijk}} + t)}{\mathcal{R}_{ijk}({B_{ijk}})}, $$

where $\mathcal{R}_{ijk}(\cdot )$ is the unconditional reliability function of component $E_{ijk}$. Without loss of generality, in this paper, the lifetime of a standard component $E_{ijk}$ is assumed to be governed by a Weibull distribution, with $\beta _{ijk}$ and $\eta _{ijk}$ being its respective shape and scale parameters. Therefore, its unconditional reliability function is:

3 $$ \mathcal{R}_{ijk}(t)=\exp \left [-\left (\frac{t}{\eta _{ijk}}\right )^{ \beta _{ijk}} \right ]. $$

For sensor-monitored components, a common set of *S* sensors is available to monitor components’ degradations, where new sensor measurements are recorded after the completion of each operating cycle. For a given component $E_{ijk}$ that has operated for $B_{ijk}$ cycles, the overall degradation measurement can be stored in a matrix $\mathbf{G}_{ijk}$ as follows:

$$ \mathbf{G}_{ijk} = \begin{bmatrix} \pmb{g}^{1} \\ \pmb{g}^{2} \\ \vdots \\ \pmb{g}^{{B_{ijk}}} \end{bmatrix} = \begin{bmatrix} g^{1}_{1} &g^{1}_{2} & \ldots &g^{1}_{S} \\ g^{2}_{1} &g^{2}_{2} & \ldots &g^{2}_{S} \\ \vdots &\vdots & & \vdots \\ g^{{B_{ijk}}}_{1} &g^{{B_{ijk}}}_{2} & \ldots &g^{{B_{ijk}}}_{S} \end{bmatrix} , $$

where $\pmb{g}^{c}$ is a vector of all sensor measurements collected during cycle *c* ($c=1,\dots ,{B_{ijk}}$). To compute the reliability of such components, a DNN with MCD is employed. The DNN, denoted as $f(\cdot ; \theta )$, is parameterized by weights and biases *θ* and aims to learn a mapping between current sensor measurements and the true RUL. To learn this mapping, the network is trained on historical degradation data, where the true RUL values are known. Let the training set be defined as $\{(\mathbf{G}_{\lambda}, RUL_{\lambda})\}_{\lambda =1}^{\Lambda}$, where $\mathbf{G}_{\lambda}$ is a matrix of historical sensor recordings, and $RUL_{\lambda}$ is the true remaining lifetime. The training process involves identifying the set of learnable weights and biases *θ* that minimize the following loss function:

4 $$ \min _{\theta} \quad \Xi = \frac{1}{\Lambda} \sum _{\lambda =1}^{ \Lambda}\left (RUL_{\lambda} - f(\mathbf{G}_{\lambda}; \theta ) \right )^{2} + \mu \cdot \Big|\Big|\theta \Big|\Big|^{2}_{2}. $$

The loss function being minimized is the mean squared error with $L_{2}$ regularization, a typical loss function used for regression tasks. Once trained, the model is capable of predicting the RUL of components currently in operation. Let $E_{ijk}$ be a sensor-monitored component with sensor measurements $\mathbf{G}_{ijk}$, the predicted RUL can then be obtained as $\widehat{RUL}_{ijk}=f(\mathbf{G}_{ijk}; \theta )$. If the trained model were capable of predicting the RUL with no error, then the probability of component $E_{ijk}$ completing the upcoming mission would be known with certainty. However, in practice, developing a perfect RUL predictive model is not possible due to model limitations, noisy data, and other factors. To account for uncertainty in the prediction, MCD is implemented. Typically, dropout is a regularization technique that is applied only during the training phase of a neural network. MCD is an approach that extends dropout to the inference phase [40]. During inference, rather than using the trained model to make a single prediction, MCD involves performing Ω forward passes with dropout applied each time. This produces Ω different RUL predictions. Let $\left (\widehat{\text{RUL}}^{1}_{ijk}, \ldots , \widehat{\text{RUL}}^{ \Omega}_{ijk}\right )$ represent the Ω RUL predictions obtained for component $E_{ijk}$. Accordingly, the reliability of component $E_{ijk}$ after completing ${B_{ijk}}$ cycles is computed as follows:

5 $$R_{ijk}^{G}(t|G_{ijk})=\frac{1}{\Omega }\sum_{\omega =1}^{\Omega }1\left({\hat{\text{RUL}}}_{ijk}^{\omega }>t\right),$$

where 1(⋅) is the indicator function taking a value of 1 if the condition in the brackets is true, and 0 otherwise. Given the individual component reliabilities, the reliability of subsystem $j \in \mathcal{J}$ of system $i \in \mathcal{I}$ is given by (6) as its configuration is parallel:

6 $$\begin{aligned} \mathcal{R}_{ij}(t)={}&1- \prod _{k \in \mathcal{K}^{\text{s}}_{j}} \left (1- \mathcal{R}_{ijk}\left (t|{B_{ijk}}\right ) \right ) \\ &{} \cdot \prod _{k \in \bar{\mathcal{K}}^{\text{s}}_{j}} \left (1- \mathcal{R}^{G}_{ijk}\left (t|\mathbf{G}_{ijk}\right ) \right ). \end{aligned}$$

### Imperfect maintenance model

During the intermission break, each failed component $E_{ijk}$ can be subjected to a CM of level $l \in \{0, \ldots , L_{ijk} \}$. The lowest maintenance level $l = 0$ corresponds to the “Do-nothing” case, while the highest level $l = L_{ijk}$ corresponds to the perfect corrective repair or “as-good-as-new” (AGAN) case, and level $l = 1$ corresponds to minimal repair, which brings the component to an “as-bad-as-old” (ABAO) condition. Intermediate values of *l* ($1 < l < L_{ijk}$) represent imperfect maintenance (IM) actions that bring the component back to a condition between AGAN and ABAO. Similarly, a working component can be subjected to a PM of level $l \in \{0, \ldots , L_{ijk} \}$. PM levels $l = 0$ and $l = L_{ijk}$ correspond to the “Do-nothing” and perfect preventive maintenance (AGAN) cases, respectively. Following the work by [16], there is no minimal repair equivalent for a PM, and the maintenance option $l = 1$ is assumed to be equivalent to the “Do-nothing” case. Intermediate values of *l* $(0 < l < L_{ijk})$ represent imperfect maintenance actions.

For standard components, the age reduction approach is used to model the impact of maintenance actions [41]. When component $E_{ijk}$ is subjected to maintenance level *l*, its age ${B_{ijk}}$ is reduced by an age reduction factor, thereby increasing its reliability. The age reduction coefficients are defined separately for corrective and preventive maintenance actions as follows:

- Corrective maintenance: the age reduction factor is $\mu ^{c}_{ijkl}$, where $0 \leq \mu ^{c}_{ijkl} \leq 1$, and the maintenance action requires a duration of $t^{c}_{ijkl}$.
- Preventive maintenance: the age reduction factor is $\mu ^{p}_{ijkl}$, where $0 \leq \mu ^{p}_{ijkl} \leq 1$, and the maintenance action requires a duration of $t^{p}_{ijkl}$.

The time required to perform maintenance level *l* on standard component $E_{ijk}$ can be formulated as:

7 $$ t_{ijkl}=t^{p}_{ijkl} \cdot {u_{ijk}} + t^{c}_{ijkl} \cdot \left (1 - {u_{ijk}}\right ), $$

and the reliability $r_{ijklm}$ of component $E_{ijk}$ after maintenance, given that maintenance level *l* has been selected and system *i* has been assigned to mission *m*, is defined as:

8 $$ r_{ijklm} = \mathcal{R}_{ijk}(U_{m}|{A_{ijk}}) \cdot v_{ijkl}, $$

where $v_{ijkl}=1(u_{ijk}=1\vee l>0)$ is the status of component $E_{ijk}$ at the end of the break, and ${A_{ijk}}$ is the effective age of $E_{ijk}$ at the end of the break. This quantity is defined as:

9 $$ {A_{ijk}} = {B_{ijk}} \, \left ({ u_{ijk}} \cdot \mu ^{p}_{ijkl} + (1-{u_{ijk}}) \cdot \mu ^{c}_{ijkl} \right ). $$

By analogy to the age reduction factor used to model IM, proposed by [41], we propose an RUL increase factor $\Delta _{ijkl}$ to model IM for sensor-monitored components. When the sensor-monitored component $E_{ijk}$ is subjected to maintenance level *l*, its RUL is extended by $\Delta _{ijkl}$. The factor $\Delta _{ijkl}$ increases with respect to the maintenance levels. Thus, the reliability $r_{ijklm}$ of component $E_{ijk}$, given that maintenance level *l* has been selected and system *i* has been assigned to mission *m*, is defined as:

10 $$r_{ijklm}=\frac{1}{\Omega }\sum_{\omega =1}^{\Omega }1\left({\hat{\text{RUL}}}_{ijk}^{\omega }+\Delta_{ijkl}>U_{m}\right).$$

In practice, a dataset containing sensor data across multiple operating cycles of a machine undergoing maintenance can be used with machine learning techniques to predict how those actions influence RUL. By analyzing historical sensor trends before and after maintenance, such models can provide data-driven estimates of $\Delta _{ijkl}$. The time required to perform maintenance level *l* on sensor-monitored component $E_{ijk}$ also follows Equation (7).

## The deterministic formulation

The fleet of systems under study is assumed to have just completed a mission and is entering a maintenance break. Due to limited resources, maintenance actions can be performed only on a subset of components across all systems. The goal of the proposed optimization model is to jointly select the components to be maintained, determine the maintenance levels for the selected components, assign repairpersons to maintenance tasks, and assign systems to mission types so that the total expected cost $\mathcal{Z}$ is minimized. The resulting optimization problem is formulated as a mixed-integer nonlinear program (MINLP) as follows:

11a $$\begin{aligned} \min \,\,\mathcal{Z} = &\sum _{m \in \mathcal{M}} \rho _{m} \left (1 - \chi _{m}\right ) \\ &{}+ \sum _{i \in \mathcal{I}} \sum _{j \in \mathcal{J}} \sum _{k \in \mathcal{K}_{j}}\sum _{l \in \mathcal{L}_{ijk}} \sum _{q \in \mathcal{Q}} c^{v} \, t_{ijkl} \, x_{ijklq}+ \sum _{q \in \mathcal{Q}}c^{f}\,\gamma _{q} \end{aligned}$$

11b $$\begin{aligned} \text{s.t.}\,\,&\sum _{m \in \mathcal{M}}z_{im} \leq 1, \qquad \forall i \in \mathcal{I}, \end{aligned}$$

11c $$\begin{aligned} & \sum _{i \in \mathcal{I}}z_{im} \geq \zeta _{m} \, \chi _{m}, \qquad \forall m \in \mathcal{M}, \end{aligned}$$

11d $$\begin{aligned} & \sum _{l\in \mathcal{L}_{ijk}}\sum _{q \in \mathcal{Q}} x_{ijklq} = 1, \qquad \forall i \in \mathcal{I}, \forall j \in \mathcal{J}, \forall k \in \mathcal{K}_{j}, \end{aligned}$$

11e $$\begin{aligned} &1 - \prod _{k \in \mathcal{K}_{j}} \left (1 - \sum _{l \in \mathcal{L}_{ijk}}\sum _{q \in \mathcal{Q}}x_{ijklq} \,r_{ijklm} \right ) \\ &\quad \geq R^{\text{min}}_{jm} \cdot z_{im}, \quad \forall i, \forall j, \forall m, \end{aligned}$$

11f $$\begin{aligned} &\sum _{i \in \mathcal{I}}\sum _{j \in \mathcal{J}}\sum _{k \in \mathcal{K}_{j}}\sum _{l \in \mathcal{L}_{ijk}\setminus \{0\}} x_{ijklq} \, t_{ijkl} \leq D \, \gamma _{q}, \quad \forall q \in \mathcal{Q}, \end{aligned}$$

11g $$\begin{aligned} & \chi _{m} \in \{0, 1 \}, \end{aligned}$$

11h $$\begin{aligned} & \gamma _{q} \in \{0, 1 \}, \end{aligned}$$

11i $$\begin{aligned} & z_{im} \in \{0, 1 \}, \end{aligned}$$

11j $$\begin{aligned} & x_{ijklq} \in \{0, 1 \}. \end{aligned}$$

The objective function (11a) minimizes the total expected cost. The first term is the cost associated with unselected mission types, and the second and third terms account for the cost of performing the selected maintenance actions and the fixed cost of hiring the necessary repairpersons. Constraints (11b) ensure that each system is assigned to no more than one mission type, while Constraints (11c) enforce the requirement that a minimum number of systems must be assigned to a mission type to be successful and thus not incur the penalty cost. Constraints (11d) enforce a single maintenance action to be selected for each component. Constraints (11e) ensure that if a system is assigned to a mission type, the reliability of the required subsystems must exceed a defined threshold (*i.e.,* required minimum reliability). Finally, Constraints (11f) guarantee that the selected maintenance actions can be performed within the break duration, and Constraints (11g)-(11j) are binary variable restrictions.

The above formulation is challenging to solve optimally due to the nonlinear reliability expression in Constraints (11e). However, these Constraints (11e) can equivalently be reformulated as linear constraints as shown through the following transformation:

$$\begin{aligned} &\prod _{k \in \mathcal{K}_{j}} \left (1 - \sum _{l \in \mathcal{L}_{ijk}} \sum _{q \in \mathcal{Q}}x_{ijklq} \cdot r_{ijklm} \right ) \\ &\quad \leq 1 - R^{ \text{min}}_{jm} \cdot z_{im},\quad {\forall i \in \mathcal{I}, \forall m \in \mathcal{M}, \forall j \in \mathcal{J}_{m}}, \\ &\ln \left (\prod _{k \in \mathcal{K}_{j}} \left (1 - \sum _{l \in \mathcal{L}_{ijk}}\sum _{q \in \mathcal{Q}}x_{ijklq} \cdot r_{ijklm} \right )\right ) \\ &\quad \leq \ln \left (1 - R^{\text{min}}_{jm} \cdot z_{im} \right ),\quad {\forall i \in \mathcal{I}, \forall m \in \mathcal{M}, \forall j \in \mathcal{J}_{m}}, \\ &\sum _{k \in \mathcal{K}_{j}}\ln \left (1 - \sum _{l \in \mathcal{L}_{ijk}} \sum _{q \in \mathcal{Q}}x_{ijklq} \cdot r_{ijklm} \right ) \\ &\quad \leq \ln \left (1 - R^{\text{min}}_{jm} \cdot z_{im}\right ),\quad {\forall i \in \mathcal{I}, \forall m \in \mathcal{M}, \forall j \in \mathcal{J}_{m}}. \end{aligned}$$

Due to the binary nature of the decision variables $x_{ijklq}$ and $z_{im}$, they can be factored out of the logarithm expressions, leading to the linearization of Constraints (11e) as:

12 $$\begin{aligned} &\sum _{k \in \mathcal{K}_{j}}\sum _{l \in \mathcal{L}_{ijk}}\sum _{q \in \mathcal{Q}}\ln \left (1 - r_{ijklm} \right ) \cdot x_{ijklq} \\ &\quad \leq \ln \left (1 - R^{\text{min}}_{jm}\right )\cdot z_{im}, \quad \forall i, \forall j, \forall m. \end{aligned}$$

The resulting mixed-integer linear program (MILP) comprises $M + Q + I \times Q + Q \times \sum _{i \in \mathcal{I}} \sum _{j \in \mathcal{J}}\sum _{k \in \mathcal{J}_{j}}L_{ijk}$ binary decision variables and a set of constraints that increase quickly with the number of components, systems, and mission types. Although the model is computationally demanding, its ability to solve practical-sized problems to optimality is demonstrated in the numerical experiments. In the following section, the methods used to deal with uncertainties in maintenance and break durations are presented and fully discussed.

## Handling uncertainty in maintenance and break durations

Variability and uncertainty in maintenance durations are inevitable due to factors such as the operating environment, the complexity of repair tasks, and the skill level of repair personnel. Furthermore, in many applications, it would be difficult to precisely forecast the exact duration of the maintenance break. For example, in military aircraft fleets, the unexpected start of the next mission/battle introduces uncertainties in the available maintenance time window [9]. Ignoring these potential uncertainties can result in a lack of readiness and exposure to risk. Rather than treating maintenance and break durations as deterministic parameters, in the present work, these durations are considered as stochastic quantities with known probability distributions. Thus, in the following, the time required to perform maintenance level *l* on component $E_{ijk}$ is represented by the random variable $\tilde{t}_{ijkl}$ with $\mathbb{E}[\tilde{t}_{ijkl}]$ as its expectation, while the uncertain break duration is represented by the random variable *D̃*. The stochastic version of the proposed FSMP is then formulated using chance constraints as follows:

13a $$\begin{aligned} \min \,\, \mathcal{Z} = {}&\sum _{m \in \mathcal{M}} \rho _{m} \left (1 - \chi _{m}\right ) \\ &{}+ \sum _{i \in \mathcal{I}} \sum _{j \in \mathcal{J}} \sum _{k \in \mathcal{K}_{j}}\sum _{l \in \mathcal{L}_{j}} \sum _{q \in \mathcal{Q}}c^{v}\, \mathbb{E}[\tilde{t}_{ijkl}] \, x_{ijklq} \\ &{} + \sum _{q \in \mathcal{Q}} c^{f} \,\gamma _{q} \end{aligned}$$

13b $$\begin{aligned} \;\text{s.t.} \quad &\mathbb{P}\left (\sum _{i \in \mathcal{I}}\sum _{j \in \mathcal{J}}\sum _{k \in \mathcal{K}_{j}}\sum _{l \in \mathcal{L}_{j} \setminus \{0\}} \tilde{t}_{ijkl}\,x_{ijklq} \leq \tilde{D} \, \gamma _{q} \right ) \\ &\quad \geq 1 - \alpha , \quad \forall q \in \mathcal{Q}, \\ &\text{(11b)}-\text{(11d)}, \\ &\text{(12)}, \\ &\text{(11g)} - \text{(11j)}. \end{aligned}$$

The chance-constrained formulation above ensures that the total maintenance time for each repairperson $q \in \mathcal{Q}$ does not exceed the allocated break duration with a specified confidence level $1-\alpha $. Parameter *α* is the confidence level specified as the risk measure to model the decision makers’ risk preferences regarding the randomness of the maintenance time and the break duration. The remaining constraints are those previously described.

Let $\mathbf{x_{q}}$ denote the vector of all maintenance actions assigned to repairperson *q*. For such a vector, we associate the random variable $Y_{q}$ defined as:

14 $$ Y_{q}:=\sum _{i \in \mathcal{I}}\sum _{j \in \mathcal{J}}\sum _{k \in \mathcal{K}_{j}}\sum _{l \in \mathcal{L}_{ijk} \setminus \{0\}} \tilde{t}_{ijkl}\,x_{ijklq} - \tilde{D}. $$

If furthermore we let $F_{Y_{q}}(y) = \mathbb{P}\left (Y_{q} \leq y\right )$ be the cumulative distribution function (CDF) of $Y_{q}$, it follows that the maintenance task assignment $\mathbf{x_{q}}$ to repairperson *q* satisfies the chance constraints of (13b) if $F_{Y_{q}}(0) \geq 1 - \alpha $. However, $Y_{q}$ is the sum of several random variables, making the computation of $F_{Y_{q}}(y)$ intractable analytically due to the required multi-dimensional integral convolutions. To address this challenge, two well-known approximation techniques are employed: Sample Average Approximation (SAA) and Conditional Value-at-Risk (CVaR). These techniques are discussed in detail in the following subsections.

### Sample average approximation

To convert the probabilistic constraints (13b) into deterministic and tractable forms, SAA is considered first. This technique approximates the original constraint using a finite set of *N* sampled scenarios as follows:

15 $$\begin{aligned}& \frac{1}{N}\sum _{n=1}^{N}\mathbb{I}\left (\sum _{i \in \mathcal{I}} \sum _{j \in \mathcal{J}}\sum _{k \in \mathcal{K}_{j}}\sum _{l \in \mathcal{L}_{ijk} \setminus \{0\}}t_{ijkl, n} \, x_{ijklq} - D_{n} \, \gamma _{q} \right ) \\& \quad \leq \alpha , \quad \forall q \in \mathcal{Q}, \end{aligned}$$

where $t_{ijkl,n}$ and $D_{n}$ are the maintenance action duration and break duration of the $n^{\text{th}}$ realization of the random parameters $\tilde{t}_{ijkl}$ and *D̃*. $\mathbb{I}(\cdot )$ is the indicator function, which equals 1 if the expression in the brackets is positive, and 0 otherwise. By introducing binary variables $y_{q,n}$ to indicate whether the constraint is violated for each realization *n*, the SAA constraints can be enforced as:

16a $$\begin{aligned} &\sum _{i \in \mathcal{I}}\sum _{j \in \mathcal{J}}\sum _{k \in \mathcal{K}_{j}}\sum _{l \in \mathcal{L}_{ijk} \setminus \{0\}}t_{ijkl, n} \, x_{ijklq} - D_{n} \, \gamma _{q} \\ &{} \quad \leq M \, y_{q,n}, \quad \forall q \in \mathcal{Q}, \forall n \in \{1, \ldots , N\}, \end{aligned}$$

16b $$\begin{aligned} &\sum _{n=1}^{N}y_{q,n} \leq \alpha \, N, \quad \forall q \in \mathcal{Q}, \end{aligned}$$

16c $$\begin{aligned} &y_{q,n} \in \{0, 1\}. \end{aligned}$$

The SAA approach is straightforward to implement but requires a significant number of samples *N* to provide good approximations. Another limitation of this method is the large number of additional binary variables it introduces. Specifically, the formulation requires $Q \times N$ additional binary variables.

### Conditional value-at-risk approximation

An alternative to the SAA method is the CVaR approximation, which provides a conservative reformulation of the chance constraints (13b). This method indeed transforms the probabilistic constraints into deterministic approximations. When the number of realizations *N* of the random parameters is finite, the probabilistic constraints (13b) are approximated using the CVaR constraints:

17 $$\begin{aligned}& \inf _{t_{q}}\Biggl\{ t_{q} + \alpha ^{-1}\sum _{n=1}^{N}p_{n} \left [ \sum _{i \in \mathcal{I}}\sum _{j \in \mathcal{J}}\sum _{k \in \mathcal{K}_{j}}\sum _{l \in \mathcal{L}_{ijk} \setminus \{0\}} t_{ijkl, n}\,x_{ijklq} \right. \\& \quad {}-\left. D_{n} \, \gamma _{q} - t_{q} \right ]_{+} \Biggl\} \leq 0, \quad \forall q \in \mathcal{Q}, \end{aligned}$$

where $[\nu ]_{+}=\max (\nu ,0)$ denotes the positive part of a real number *ν*, and $t_{ijkl, n}$ and $D_{n}$ are the *n*th samples of $t_{ijkl}$ and *D* respectively, and $p_{n}$ denotes the probability of observing sample *n*. By introducing auxiliary variables $u_{q,n}$ and $t_{q}$, the constraints can be reformulated as:

18a $$\begin{aligned} &t_{q} + \alpha ^{-1} \sum _{n=1}^{N}p_{n} \, u_{q,n} \leq 0, \qquad \forall q \in \mathcal{Q}, \end{aligned}$$

18b $$\begin{aligned} &u_{q,n} \geq \sum _{i \in \mathcal{I}}\sum _{j \in \mathcal{J}_{i}} \sum _{k \in \mathcal{K}_{j}}\sum _{l \in \mathcal{L}_{ijk} \setminus \{0\}}t_{ijkl, n}\,x_{ijklq} \\ &\hphantom{u_{q,n} \geq}{} - D_{n} \, \gamma _{q} - t_{q}, \quad \forall q \in \mathcal{Q}, \forall n \in \{1, \ldots , N\}, \end{aligned}$$

18c $$\begin{aligned} &u_{q,n} \geq 0, \qquad \forall q \in \mathcal{Q}, \forall n \in \{1, \ldots , N\}. \end{aligned}$$

Regarding the derivation of the CVaR constraints, the reader can find more details in [42]. The CVaR approximation is more conservative than SAA; however, it offers computational efficiency as only continuous variables are introduced.

## Comparison of SAA and CVaR

**Figure 1:**
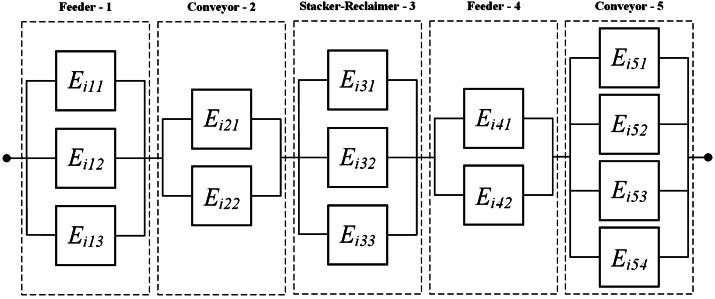
Real-world coal transportation system

This section aims to provide a comparison between the SAA and CVaR approximation methods and demonstrate the real-world applicability of the proposed optimization model. For this purpose, a fleet composed of $I=2$ identical coal transportation systems that supply a boiler in a plant is considered. This example is inspired by the case study considered in multiple SMP papers [9, 13, 43]. Figure 1 displays the RBD of each system in this fleet, comprising $J=5$ subsystems: two conveyors, two feeders, and a stacker-reclaimer. All components are standard and Table 1 gives their corresponding lifetime distribution parameters as well as their respective initial ages and states. A list of four potential maintenance levels is available, including do-nothing, minimal repair (for failed components only), imperfect maintenance, and perfect repair (*i.e.,* renewal). All maintenance durations are assumed to follow a truncated normal distribution, where the respective mean *μ*, standard deviation *σ*, minimum *a* and maximum *b* values are reported in Table 2. The break duration *D* follows a uniform distribution in the range $[10, 20]$, *i.e.,* $D \sim \mathcal{U}(10, 20) $, and Table 3 provides a detailed description of the different mission types considered, including the penalty for unassigned mission, duration, and other relevant input data. Two ($Q=2$) repairpersons are available to carry out the maintenance tasks, and similar to [13], the fixed and variable costs are set to $c^{f}=\$25$ and $c^{v}=\$8.5$, respectively. The proposed optimization model is implemented in Python and solved using Gurobi 11.0. A time limit of 600 seconds is set for each instance solved.

**Table 1:** Component data for standard components

	Initial age $(\varphi _{ijk})$	Initial state $(u_{ijk})$	Weibull shape parameter $(\beta _{ijk})$	Weibull scale parameter $(\eta _{ijk})$
$E_{1,1,1}$	35	1	1.5	150
$E_{1,1,2}$	24	0	2.4	228
$E_{1,1,3}$	45	0	1.6	168
$E_{1,2,1}$	35	0	2.6	240
$E_{1,2,2}$	28	1	1.8	168
$E_{1,3,1}$	36	1	2.4	204
$E_{1,3,2}$	44	0	2.5	156
$E_{1,3,3}$	28	0	2.0	168
$E_{1,4,1}$	38	1	1.2	156
$E_{1,4,2}$	15	0	1.4	210
$E_{1,5,1}$	30	0	2.8	240
$E_{1,5,2}$	22	1	1.5	210
$E_{1,5,3}$	38	1	2.4	180
$E_{1,5,4}$	35	0	2.2	270
$E_{2,1,1}$	35	0	1.5	150
$E_{2,1,2}$	38	1	2.4	228
$E_{2,1,3}$	22	1	1.6	168
$E_{2,2,1}$	30	1	2.6	240
$E_{2,2,2}$	15	0	1.8	168
$E_{2,3,1}$	38	1	2.4	204
$E_{2,3,2}$	28	0	2.5	156
$E_{2,3,3}$	44	0	2.0	168
$E_{2,4,1}$	36	1	1.2	156
$E_{2,4,2}$	28	1	1.4	210
$E_{2,5,1}$	35	0	2.8	240
$E_{2,5,2}$	45	0	1.5	210
$E_{2,5,3}$	24	0	2.4	180
$E_{2,5,4}$	35	1	2.2	270

**Table 2:** Maintenance duration parameters for different components and maintenance levels

$E_{ijk}$	$t^{p}_{ij2}$				$t^{p}_{ij3}$				$t^{c}_{ij1}$				$t^{c}_{ij2}$				$t^{c}_{ij3}$			
	*μ*	*σ*	*a*	*b*	*μ*	*σ*	*a*	*b*	*μ*	*σ*	*a*	*b*	*μ*	*σ*	*a*	*b*	*μ*	*σ*	*a*	*b*
$E_{i11}$	1.33	0.27	0.66	2.65	2.64	0.53	1.32	5.29	1.11	0.22	0.56	2.22	2.44	0.49	1.22	4.89	4.6	0.92	2.3	9.2
$E_{i12}$	1.31	0.26	0.65	2.61	2.65	0.53	1.33	5.3	1.02	0.2	0.51	2.04	2.09	0.42	1.04	4.18	6.3	1.26	3.15	12.59
$E_{i13}$	1.26	0.25	0.63	2.51	2.4	0.48	1.2	4.79	1.11	0.22	0.56	2.23	2.1	0.42	1.05	4.2	5.31	1.06	2.65	10.62
$E_{i21}$	1.7	0.34	0.85	3.41	2.77	0.55	1.38	5.54	0.87	0.17	0.5	1.73	2.8	0.56	1.4	5.6	5.55	1.11	2.77	11.1
$E_{i22}$	1.4	0.28	0.7	2.79	2.44	0.49	1.22	4.89	1.18	0.24	0.59	2.35	2.8	0.56	1.4	5.6	4.76	0.95	2.38	9.53
$E_{i31}$	1.7	0.34	0.85	3.4	2.71	0.54	1.36	5.43	0.94	0.19	0.5	1.89	2.78	0.56	1.39	5.55	6.21	1.24	3.1	12.41
$E_{i32}$	1.23	0.25	0.61	2.45	2.5	0.5	1.25	5.0	0.89	0.18	0.5	1.78	2.1	0.42	1.05	4.2	6.57	1.31	3.28	13.14
$E_{i33}$	1.56	0.31	0.78	3.12	2.81	0.56	1.4	5.61	1.16	0.23	0.58	2.33	2.29	0.46	1.14	4.57	4.89	0.98	2.45	9.79
$E_{i41}$	1.71	0.34	0.85	3.42	2.15	0.43	1.08	4.3	0.89	0.18	0.5	1.78	2.04	0.41	1.02	4.08	5.32	1.06	2.66	10.64
$E_{i42}$	1.35	0.27	0.67	2.7	2.6	0.52	1.3	5.2	0.94	0.19	0.5	1.88	2.03	0.41	1.01	4.06	4.99	1.0	2.49	9.98
$E_{i51}$	1.25	0.25	0.63	2.51	2.44	0.49	1.22	4.89	1.19	0.24	0.59	2.38	2.18	0.44	1.09	4.36	5.02	1.0	2.51	10.03
$E_{i52}$	1.69	0.34	0.84	3.38	2.19	0.44	1.09	4.37	1.1	0.22	0.55	2.19	2.94	0.59	1.47	5.88	6.53	1.31	3.27	13.07
$E_{i53}$	1.71	0.34	0.86	3.42	2.53	0.51	1.27	5.06	0.96	0.19	0.5	1.92	2.97	0.59	1.49	5.94	5.05	1.01	2.53	10.1
$E_{i54}$	1.37	0.27	0.69	2.75	2.62	0.52	1.31	5.25	0.95	0.19	0.5	1.89	2.07	0.41	1.04	4.14	6.17	1.23	3.08	12.34

**Table 3:** Mission profiles

*m*	$\rho _{m} (\$)$	$U_{m}$ (hours)	$\zeta _{m}$	$\mathcal{J}_{m}$	$\mathcal{R}_{m}$
1	1000	50	1	{1,2,3,4,5}	{0.995,0.990,0.995,0.970,0.999}
2	500	50	1	{1,2,3,4,5}	{0.995,0.990,0.995,0.970,0.999}

**Figure 2:**
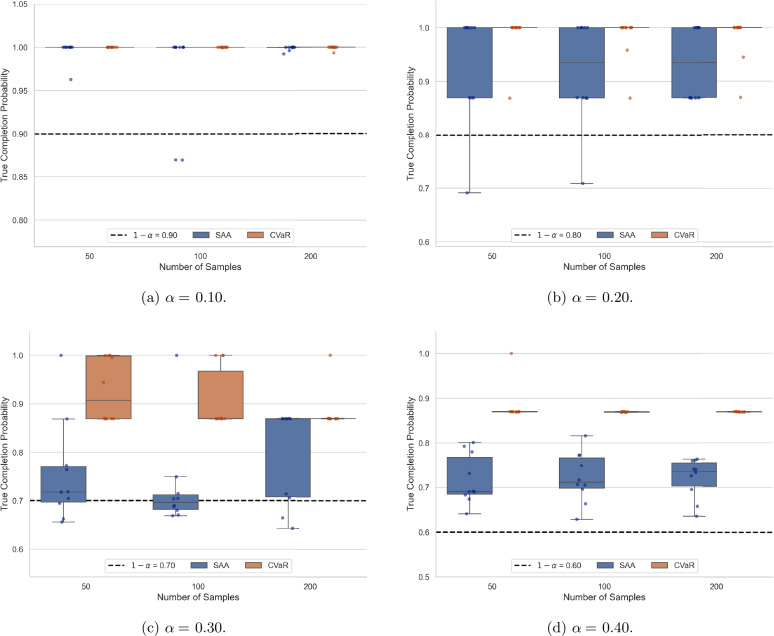
Constraint satisfaction probability for varying values of *α*

To perform the comparative analysis, we solve the problem for various service levels $1-\alpha $ and different sample sizes *N* of the random parameters. For each combination of $1-\alpha $ and *N*, the problem is solved 10 times using independently generated realizations of the random parameters. Table 4 provides the mean and standard deviation of the objective function value over the 10 runs for the SAA and CVaR approximation methods. From these results, it can be observed that for larger values of $1 - \alpha $, both SAA and CVaR tend to produce similar mean objective values, with SAA performing slightly better. For smaller values of $1-\alpha $, SAA tends to result in significantly lower mean objective function values. This is primarily because SAA assigns a system to each mission type, in contrast to the more conservative CVaR approach, which typically assigns only one system. Of course, assigning both systems requires more maintenance tasks to be performed, leading to an increase in the maintenance time per repairperson. Since the CVaR approach is inherently more conservative, it avoids such assignments to ensure that the solution remains feasible in terms of the required service level $1-\alpha $. Although SAA performs better in terms of the value of the objective function, especially for smaller values of $1-\alpha $, it is important to highlight the resulting disparity in terms of computational time and solution variability. Across all instances solved, the CVaR approximation method results in far faster solution time; this is notable for larger sample sizes and larger values of $1-\alpha $. However, the SAA approximation method introduces many additional binary decision variables, leading then to long solution times. Additionally, SAA tends to result in greater variability in objective value across the different runs, particularly at smaller sample sizes and higher confidence levels. This is evidenced by the larger standard deviations reported in Table 4. In contrast, the CVaR method provides more stable solutions with low variance. Another limitation of the SAA approach can be observed in Fig. 2, which displays the true completion probability for varying values of $1-\alpha $ and sample sizes *N*. After solving each instance, the assigned maintenance actions were used to compute the true probability that each repairperson completes their tasks within the break duration. This was estimated using Monte Carlo simulation based on the known distributions of maintenance and break durations. For each of the 10 instances solved at each combination of $1-\alpha $ and *N*, Fig. 2 reports the minimum completion probability across the two repairpersons. As shown, the SAA approach results in violations of the required service level for several of the instances solved, with true completion probabilities falling below the specified threshold $1-\alpha $. In contrast, the CVaR approach consistently produces solutions that meet or exceed the target.

**Table 4:** Comparison of SAA and CVaR

1 − *α*	*N*	SAA				CVaR			
		$\mu _{\text{obj}}$	$\sigma _{\text{obj}}$	$\mu _{\text{CPUt}}$	$\sigma _{\text{CPUt}}$	$\mu _{\text{obj}}$	$\sigma _{\text{obj}}$	$\mu _{\text{CPUt}}$	$\sigma _{\text{CPUt}}$
0.50	50	341.24	0.00	3.72	1.53	646.42	0.00	0.18	0.06
	100	341.24	0.00	7.73	6.40	615.90	91.55	0.51	0.38
	200	341.24	0.00	27.18	19.50	646.42	0.00	0.99	0.66
0.60	50	341.24	0.00	3.48	2.00	648.92	7.50	0.22	0.09
	100	341.24	0.00	5.25	2.61	646.42	0.00	0.43	0.16
	200	341.24	0.00	19.89	20.11	646.42	0.00	0.90	0.29
0.70	50	402.28	122.07	14.27	28.01	658.92	12.50	0.24	0.13
	100	371.76	91.55	65.37	178.26	653.92	11.46	0.38	0.18
	200	524.35	149.50	431.72	256.14	648.92	7.50	0.95	0.33
0.80	50	618.40	92.69	16.66	12.02	668.92	7.50	0.32	0.14
	00	616.06	91.61	461.21	208.63	668.92	7.50	0.49	0.18
	200	646.42	0.00	600.00	0.00	668.92	7.50	1.22	0.53
0.90	50	666.42	10.00	5.58	2.68	671.42	0.00	0.23	0.14
	100	663.92	11.46	32.61	33.41	671.42	0.00	0.43	0.22
	200	668.92	7.50	552.60	142.19	671.42	0.00	0.90	0.47

The overall results of this experiment demonstrate the pros and cons of both the SAA and CVaR approximation methods. Although the SAA method often yields solutions with lower objective function values, it can lead to violations of the probabilistic constraints. Additionally, it takes significant time to solve, especially as the number of random samples increases. In contrast, the CVaR approximation method consistently produces feasible solutions that often exceed the required service level, with lower solution variability and significantly faster computational time. However, due to the inherent conservatism of the CVaR approximation, it may lead to overly cautious maintenance plans that result in fewer missions being selected and completed, even in scenarios where successful completion would have been possible. Due to the significant improvement in computational time and solution variability, the CVaR approximation method is used for the case study presented in the following section.

## Case study: military aircraft fleet maintenance

**Figure 3:**
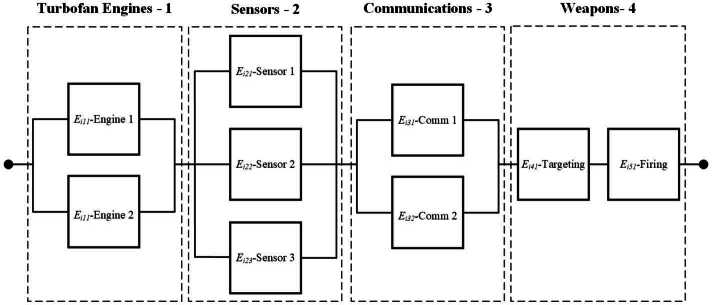
Aircraft RBD considered for the case study

This section illustrates how the proposed optimization model can deal with joint maintenance, repairpersons assignment decisions, and assignment of systems to mission types for a fleet composed of $I=4$ military aircraft. According to the reliability block diagram in Fig. 3, each aircraft is comprised of four key subsystems: propulsion (engines), sensors and communication, and combat systems (targeting, offensive weapons). It is assumed that the engines are sensor-monitored while all other components are standard with known lifetime distributions. The NASA C-MAPSS (Commercial Modular Aero-Propulsion System Simulation) dataset is used to train our DNN for the engines. This dataset provides degradation trajectories under 4 different operational conditions and fault modes for turbofan engines. Because these different sets consider different operational conditions and fault modes, they cannot be jointly studied. In this paper, the first set called FD001 which considers a single fault mode is selected. This set provides run-to-failure information for 100 engines and includes 3 operational settings and 21 sensor values for each operating cycle. The detailed description of the 21 sensor variables can be found in [44]. In total, 20,631 cycles are available for training. Each engine starts with a different degree of initial wear and manufacturing variation. The operational settings and sensor values are provided for every cycle up until engine failure. A second test set is also provided that includes truncated time series of various lengths before failure for 100 engines and actual RULs.

The engine data from FD001 is randomly assigned to aircraft engines to simulate a diverse range of initial health conditions. The engine assignment, RUL point prediction, and predicted reliability can be found in Table A.1 of the Appendix. In this Appendix, Table A.2 gives the parameters of the lifetime distributions of the standard components. Let us recall that, for the sensor-monitored engines, a single maintenance action is available, which restores the engines to the as-good-as-new state. For standard components, a list of four potential maintenance levels is available, including do-nothing, minimal repair (for failed components only), imperfect maintenance, and perfect repair. All maintenance durations are assumed to follow a Gamma distribution, where the respective shape and scale parameters $\xi _{sh}$ and $\xi _{sc}$ are reported in Table A.3 of the Appendix. Table 5 provides a detailed description of the different mission types considered, including the penalty for unassigned mission, duration, and other relevant input data. It is important to note that operational data for sensor-monitored components is recorded in cycles, while the lifetimes of standard components are measured in operating hours. Accordingly, Table 5 provides the mission durations in both operating hours and cycles.

**Table 5:** Mission profiles

*m*	Mission Description	$\rho _{m} (\$)$	$U_{m}$ (hours/cycles)	$\zeta _{m}$	$\mathcal{J}_{m}$	$\mathcal{R}_{m}$
1	Combat	80,000	2 / 10	2	{1,2,3,4,5}	{0.995,0.990,0.990,0.950,0.950}
2	Reconnaissance	60,000	5 / 25	1	{1,2,3}	{0.995,0.990,0.950}
3	Logistics Support	40,000	8 / 40	1	{1,2,3}	{0.995,0.990,0.900}

The following subsections will first present the results of the DNN with MCD for reliability prediction, followed by multiple experiments to demonstrate the impact of stochastic durations and the risk confidence level parameter *α* on the maintenance plan and the overall assignment decisions.

### Performance analysis of DNN with MCD

In this work, the deep learning architecture implemented for RUL prediction is a recurrent neural network (RNN), specifically a bi-directional long short-term memory (Bi-LSTM) network. LSTMs are a type of RNN designed to overcome the vanishing and exploding gradient problem frequently encountered in standard RNNs. Bi-LSTMs comprise two LSTM cells and have demonstrated exceptional performance in predicting remaining useful life [33, 36]. The main advantage of using Bi-LSTMs instead of standard RNNs is that they can learn the temporal relationships in the forward and backward directions simultaneously. For detailed information on the inner workings of the LSTM cells, the reader is referred to [45]. Before training the Bi-LSTM for RUL prediction, the NASA C-MAPSS FD001 dataset was pre-processed and cleaned according to the following steps:

- *Data labeling*. A column was added to both the training and testing sets that specified the RUL after completion of each cycle. This column provides the labels that will be used to train and test the models.
- *Input data scaling*. It is widely acknowledged that scaling input data for neural networks leads to more efficient training. The min-max normalization approach was used to scale the input of the DL algorithm to be in the range $[0,1]$. The min-max normalization of data is performed according to the following formula:
  19 $$ \check{x}^{i}_{j}= \dfrac{x_{ij} - x_{j}^{min}}{x_{j}^{max} - x_{j}^{min}}, $$
  where $x^{i}_{j}$ denotes the *i*th data measure of the *j*th sensor, $\check{x}^{i}_{j}$ is the scaled value of $x^{i}_{j}$, $x_{j}^{max}$ and $x_{j}^{min}$ are the respective maximum and minimum raw measurements from the *j*th sensor.
- *Data reformatting*. In preparation for fitting the Bi-LSTM, the training and testing data was reformatted into 3-dimensional tensors of the form ($N_{s}$, $N_{ts}$, $N_{f}$). $N_{s}$ refers to the number of samples, $N_{ts}$ denotes the time sequence dimension, and $N_{f}$ refers to the number of selected features.

For the Bi-LSTM, the hyperparameters proposed in [33] were adopted. The following three commonly used metrics are exploited to evaluate the proposed Bi-LSTM performance for point prediction: root mean squared error (RMSE), scoring function (SC), and accuracy (AC). Smaller values of RMSE and SC, along with higher values of AC are preferred. Assuming a total of Λ test examples with Ω predictions for each test example $\lambda \in \{1, \ldots \Lambda \}$, we define $d_{\lambda} = \left (\frac{1}{\Omega}\sum _{\omega =1}^{\Omega} \widehat{\text{RUL}}_{\lambda \omega}\right ) - \text{RUL}_{\lambda}$ as the difference between the average prediction and the target. The mathematical expression corresponding to each of the above valuation metrics is:

20 $$\begin{aligned}& \text{RMSE} = \sqrt{ \frac{\sum _{\lambda =1}^{\Lambda}\left (d_{\lambda} \right )^{2}}{\Lambda}}, \end{aligned}$$

21 $$\begin{aligned}& \text{SC} = \sum _{\lambda =1}^{\Lambda}s_{\lambda}, \quad \text{where} \quad s_{\lambda}= \textstyle\begin{cases} \exp{\left (\frac{-d_{\lambda}}{13} \right )} - 1, & \text{if $d_{\lambda} \leq 0$}, \\ \exp{\left (\frac{d_{\lambda}}{10} \right )} - 1, & \text{if $d_{\lambda} \geq 0$}, \end{cases}\displaystyle \end{aligned}$$

22 $$\begin{aligned}& \text{AC} = \frac{100}{\Lambda}\sum _{\lambda =1}^{\Lambda}a_{\lambda}, \quad \text{where} \\& \quad a_{\lambda}= \textstyle\begin{cases} 1, & \text{if $d_{\lambda} \in [-13, 10 ]$}, \\ 0, & \text{if $d_{\lambda} \notin [-13, 10 ]$}. \end{cases}\displaystyle \end{aligned}$$

The same early stopping, learning rate decay strategy, and piecewise target function proposed in [33] are also implemented. Furthermore, the impact of the dropout rate on prediction accuracy and the predicted RUL distribution is analyzed. For varying dropout rates, Table 6 displays the results obtained across multiple point prediction metrics. Interestingly, models with different dropout rates demonstrate comparable performance across the different metrics considered. However, a dropout rate of 0.2 results in the worst performance across the three metrics considered for the full dataset, likely due to overfitting to the training data. For all dropout values, it can be seen that the accuracy of the models improves significantly as the number of remaining operating cycles approaches 0. This is an important observation which indicates that the models become more accurate as the engine approaches failure. All models achieve an RMSE below 5 when considering monitoring points of 25 remaining cycles, in contrast to a significantly higher RMSE of approximately 18 when considering monitoring points of 75 remaining cycles.

**Table 6:** Impact of dropout rate on prediction accuracy

Dropout	Full dataset			Monitoring point with 75 cycles left		Monitoring point with 50 cycles left		Monitoring point with 25 cycles left	
	RMSE	SC	AC	RMSE	AC	RMSE	AC	RMSE	AC
0.2	13.10	310.24	68.00	20.14	53.49	6.12	87.89	3.46	100.00
0.3	11.71	246.15	76.00	18.21	58.14	7.00	90.91	3.85	100.00
0.4	11.56	278.07	69.00	18.76	53.49	7.27	90.91	3.46	100.00
0.5	11.74	254.53	72.00	18.47	53.49	6.70	87.87	4.23	100.00

**Figure 4:**
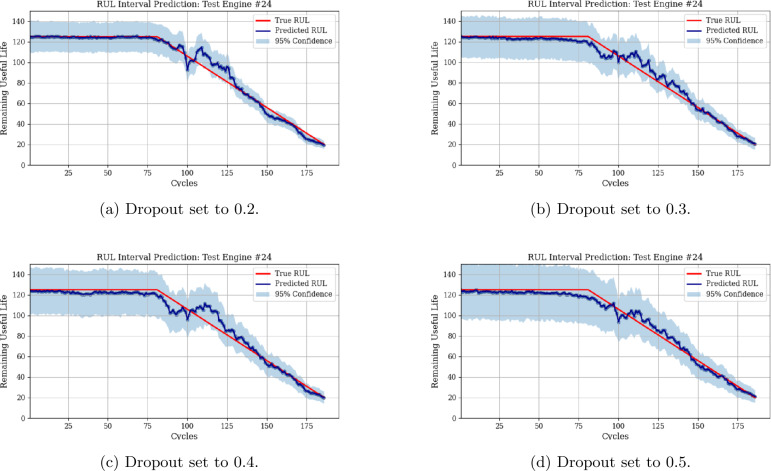
RUL interval estimate for test engine 24 for varying dropout rates

**Figure 5:**
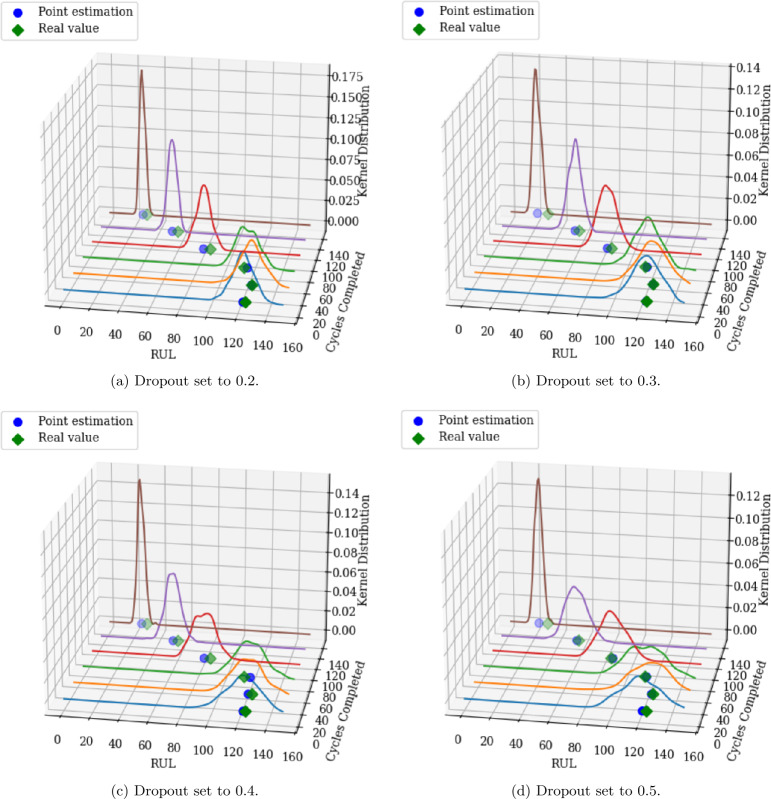
RUL distribution plots for test engine 90 for varying dropout rates

MCD is utilized in this work to estimate the uncertainty of RUL predictions. By performing multiple stochastic forward passes through the model during inference, MCD generates a distribution of predictions. Figure 4 displays the mean of the MCD predictions (blue line) and 95% confidence interval (shaded region) for test engine 24, and for varying dropout rates. As one may observe, a dropout rate of 0.2 produces narrower confidence intervals, indicating reduced uncertainty, whereas a dropout rate of 0.5 results in broader intervals. This trade-off highlights the impact of the dropout rate on uncertainty quantification, with higher dropout rates offering more robust confidence estimates. Figure 5 further emphasizes the impact of the dropout rate on the width of the predicted RUL distribution. This figure displays the kernel distribution for test engine 90 considering multiple monitoring points for varying dropout values. Since MCD is used to compute the probability of successfully completing the upcoming mission, higher dropout rates may lead to unnecessary maintenance tasks due to the wider predicted RUL distribution. In contrast, lower dropout rates may increase the risk of component failure as the predicted RUL distribution becomes narrow.

**Figure 6:**
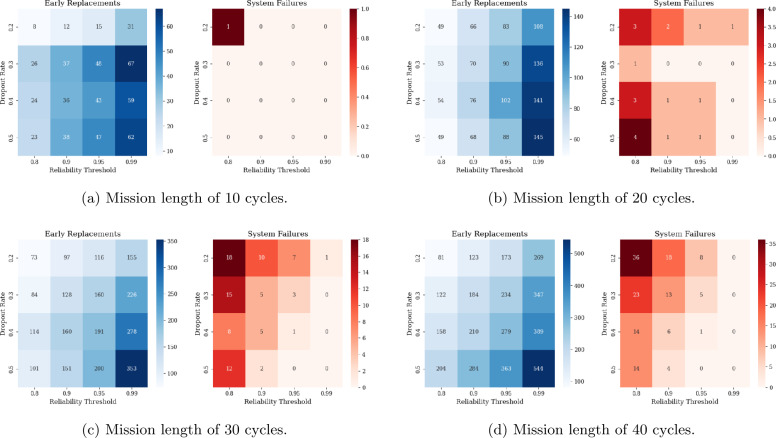
Impact of reliability threshold and dropout rate on early replacements and system failures for different mission lengths

To clearly demonstrate the impact of the dropout rate on maintenance decisions, Fig. 6 displays the number of early replacements and system failures for the engines in the CMAPSS FD001 test set. These metrics are computed as follows:

- If the predicted engine reliability is lower than the target reliability, but the engine survives the upcoming mission of duration *U* (*i.e.,* the true RUL is greater than *U*), this is counted as an early replacement.
- If the predicted engine reliability is higher than the target reliability, but the engine fails during the upcoming mission of duration *U* (*i.e.,* the true RUL is less than *U*), this is counted as a failure of the engine.

The above metrics are computed for all cycles completed across all 100 test engines and the results obtained are plotted in Fig. 6. These results highlight the trade-off between early replacements and system failures, as well as the improved prediction performance for shorter mission durations. Generally, higher dropout values result in fewer system failures, but an increase in early replacements. In the case of exceptionally high reliability targets (99%), dropout values above 0.2 completely eliminate system failures. As expected, increasing the reliability requirement leads to fewer system failures but also more early replacements. Therefore, decision-makers have to choose an appropriate balance based on risk tolerance and cost constraints. Selecting high dropout values and high reliability requirements will result in an aggressive plan that likely leads to very few failures with high maintenance costs. In contrast, riskier maintenance strategies (*i.e.*, low dropout rate and/or low reliability thresholds) will likely result in reduced maintenance costs but increased mission failure probability. For the remaining numerical experiments, a dropout rate of 0.3 is selected as it provides the fewest early replacements while ensuring no system failures.

### Impact of stochastic parameters

This set of experiments aims to demonstrate the importance of considering the stochastic nature of maintenance and break durations. In these experiments, the break duration is modeled as a truncated normal distribution with lower and upper bounds of 50 and 350 hours, respectively, a standard deviation of 50 hours, and a mean of 200 hours. There are $Q=4$ available repairpersons to carry out the selected maintenance levels, with the respective fixed and variable costs of $c^{f}=\$2500$ and $c^{v}=\$50$. Finally, the required probability of completing the selected maintenance actions is set to $1-\alpha =90\%$. The proposed optimization model is implemented in Python and solved using Gurobi 11.0. A time limit of 600 seconds is set for each instance solved.

**Figure 7:**
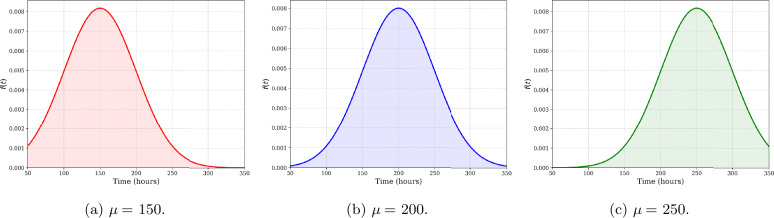
Probability density functions of the three break durations considered

**Figure 8:**
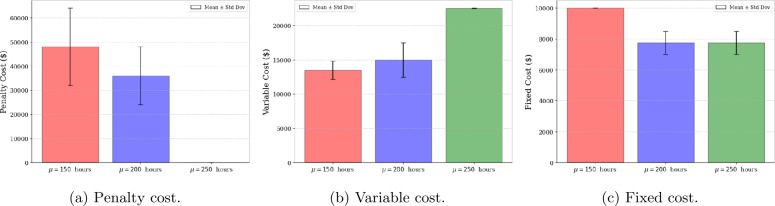
Mean and standard deviation of penalty cost, variable cost, and fixed cost for varying values of mean break duration

**Figure 9:**
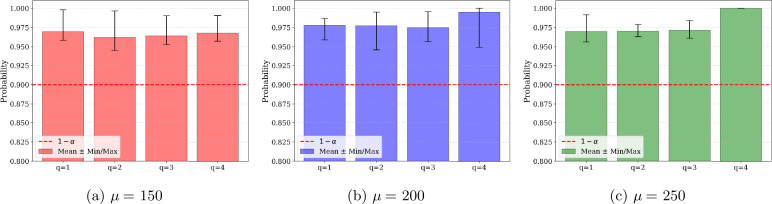
Constraint satisfaction probability for varying values of mean break duration

To illustrate the impact of the mean duration of the break on maintenance and assignment decisions, three mean break durations are considered: $\mu = 150$, $\mu =200$, and $\mu = 250$ hours. The probability density functions (*pdf*) of the three break durations are shown in Fig. 7. The number of realizations of the random maintenance and break durations generated is set to $N=500$, and the optimization model is solved 10 times for each instance to account for variability in the random variable realizations. For each value of the break mean duration, Fig. 8 displays the mean and standard deviation of the penalty cost, variable cost, and fixed cost. The results obtained show that increasing the mean break duration leads to a reduction in penalty cost, as more missions can be initiated. Indeed, a longer break duration allows for additional maintenance tasks while ensuring that the constraint satisfaction probability is met. The observed increase in average variable cost results from additional maintenance required to ensure the readiness of more aircraft, enabling the initiation of more missions. Figure 9 displays the true constraint satisfaction probability for the four available repairpersons. This probability is calculated by generating a large number of realizations for the durations of both selected maintenance actions and the break. The constraint satisfaction probability is then computed as the ratio of the number of scenarios with no constraint violations to the total number of realizations generated. It can be seen that for all mean break durations, no repairperson violates the required constraint satisfaction probability of $1-\alpha =0.9$.

To highlight the importance of accounting for the stochastic nature of maintenance and break durations, the solutions obtained using only the average values for these durations are reported in Table 7 for different mean break durations. This table gives the penalty, variable cost, fixed cost, the number of missions initiated $(M^{\ast})$, the number of repairpersons hired and utilized $(Q^{\ast})$, the probability of true constraint satisfaction for multiple repairpersons, the average overtime and the computational time. The average overtime represents the expected additional time required to complete the selected maintenance tasks. Considering only average values results in overly optimistic solutions that have low probabilities of being completed within the stochastic break duration. In some cases, the probability of completing the assigned maintenance tasks is around or even below 50%. It is also important to highlight the significant increase in average overtime when considering only the mean values of maintenance and break durations. In the context of a military aircraft fleet during wartime, requiring substantial additional time to complete maintenance tasks could delay critical operations and put lives at risk.

**Table 7:** Comparison of Deterministic and Stochastic Solutions. Mean and standard deviation (in brackets) reported for CVaR approach

		Penalty ($ × 10^3^)	Var. Cost ($ × 10^3^)	Fixed Cost ($ × 10^3^)	$M^{\ast}$	$Q^{\ast}$	Constraint Satisfaction Probability (%)				Avg. Overtime (hr)	CPU Time (s)
							*q* = 1	*q* = 2	*q* = 3	*q* = 4		
*μ* = 150	Determ.	0	22.5	7.5	3.0	3.0	50.7	53.3	49.6	–	19.79	0.1
	CVaR	48(16)	13.5(1.3)	10.0(0.0)	2.0(0.0)	4.0(0.0)	96.9(1.1)	96.2(1.7)	96.3(1.0)	96.7(1.2)	0.55(0.16)	19.7(34.7)
*μ* = 200	Determ.	0	22.5	7.5	3.0	3.0	60.0	79.0	96.5	–	15.17	0.2
	CVaR	36(12)	14.9(2.5)	7.8(0.8)	2.1(0.3)	3.1(0.3)	97.8(0.9)	97.7(1.4)	97.5(1.0)	99.5(1.5)	0.62(0.23)	107.4(129.6)
*μ* = 250	Determ.	0	22.5	7.5	3.0	2.0	56.3	76.7	–	–	17.68	0.1
	CVaR	0(0)	22.5(0)	7.8(0.8)	3.0(0.0)	3.0(0.0)	97.0(1.0)	97.0(0.6)	97.1(0.8)	–	0.76(0.14)	13.9(2.3)

**Figure 10:**
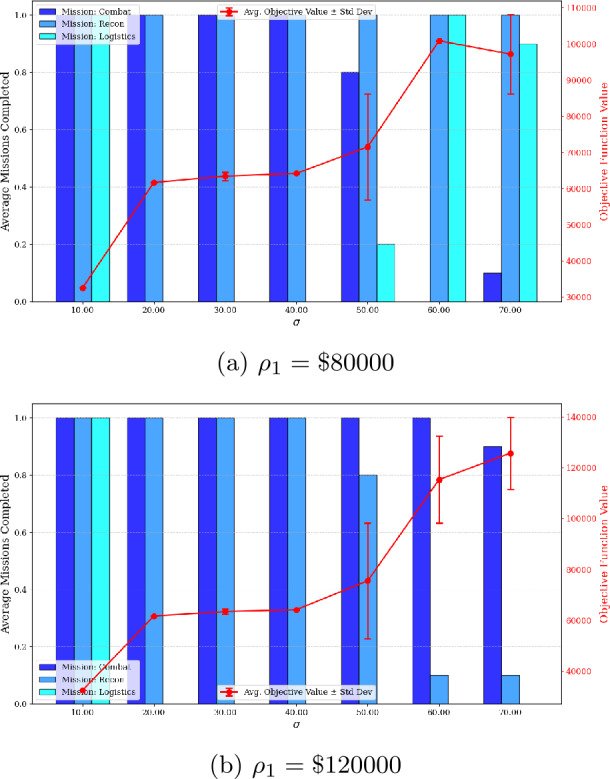
Impact of standard deviation of break duration on mission selection probability and objective function value

Finally, the impact of the standard deviation of the break duration on maintenance plan, system-to-mission assignment, and the objective function value is studied using a truncated normal distribution with a mean of 150 hours. The standard deviation is varied from 10 to 70 hours and the results are displayed in Fig. 10. This figure illustrates how the probability of selecting each mission type changes over the 10 optimization runs as the standard deviation of the break increases. The results are reported for two values of $\rho _{1}$, the penalty assigned to the combat mission. The bar chart represents the probability of initiating combat, reconnaissance, and logistics missions, while the line plot shows the corresponding objective function value. Generally, a decreasing trend in the objective function value can be seen as the break duration standard deviation increases. A higher standard deviation of the break duration limits the number of maintenance actions each repairperson can perform while ensuring the desired constraint satisfaction probability. Consequently, more repairpersons must be hired, increasing fixed costs, or fewer mission types can be selected. Another interesting observation is the shift in mission type selection as the standard deviation increases. For $\rho _{1} = \$80{,}000$, the probability of selecting the combat mission decreases as the standard deviation increases. Although the combat mission carries the highest penalty, it requires two aircraft for successful completion and also requires the functionality of the weapons subsystem. As uncertainty increases, the model favors selecting the reconnaissance and logistics support missions, whose combined penalty exceeds that of the combat mission alone while requiring the same number of operational systems. However, when $\rho _{1} = \$120{,}000$, the model tends to select the combat mission, as its penalty surpasses the combined penalty of the other two mission types.

### Impact of constraint satisfaction probability

**Figure 11:**
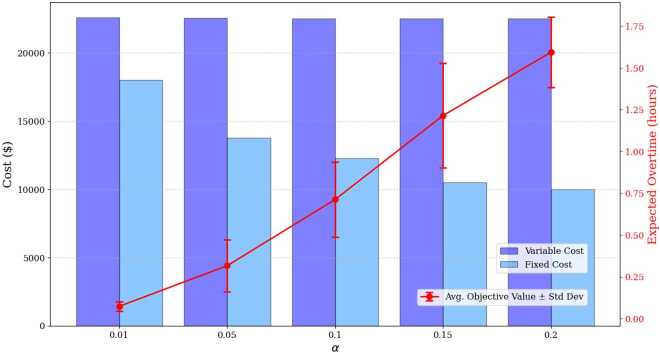
Average variable cost, fixed cost, and overtime for varying values of *α*

**Figure 12:**
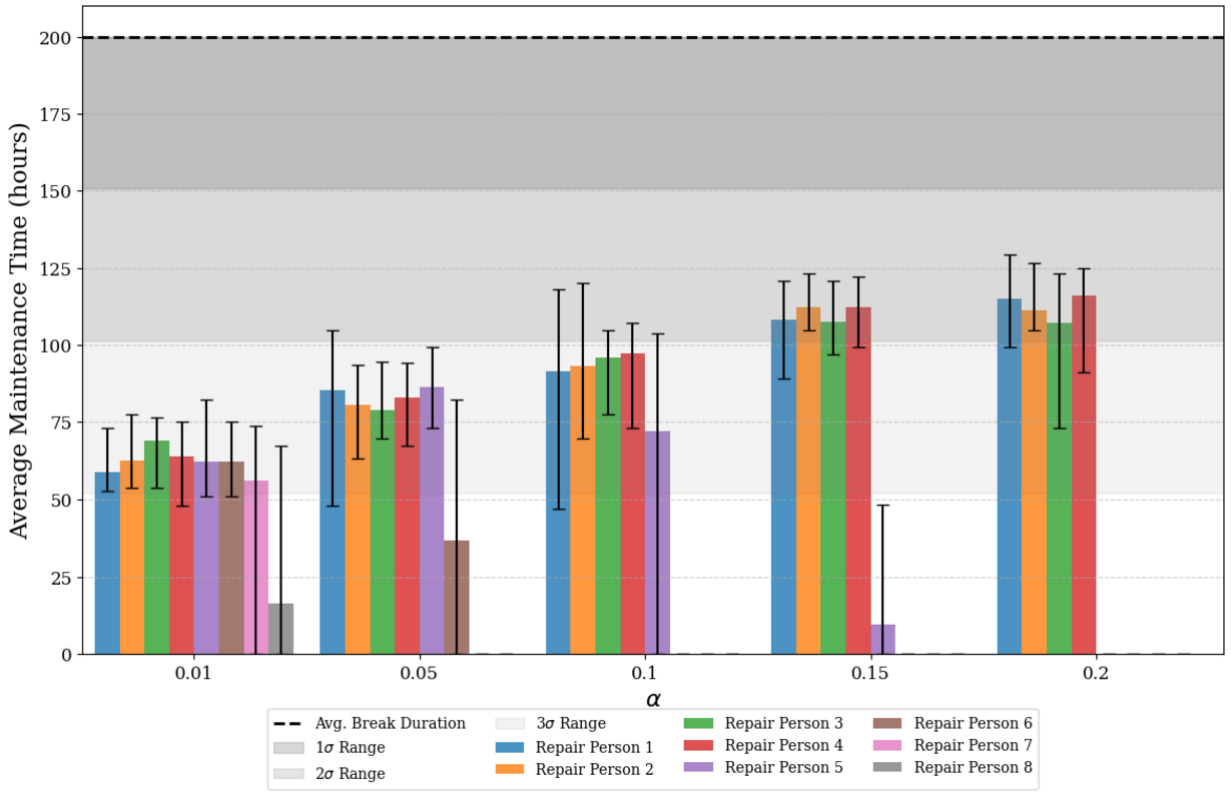
Average maintenance durations for varying values of *α*

This final experiment aims to explore the impact of the constraint satisfaction probability $1-\alpha $. To do so, a truncated normal distribution with mean 200 hours, standard deviation of 50 hours, and lower and upper bounds of 50 and 350 hours is once again considered. In this set of experiments, $Q=8$ repairpersons are available to carry out the selected maintenance actions, while all other input data remain unchanged. The problem is solved for varying values of *α* and the results are displayed in Fig. 11. For all values of *α*, all mission types are selected; however, more repairpersons are required to perform the necessary maintenance tasks for smaller values of *α*. This is because smaller values of *α* imply a higher required probability of constraint satisfaction, meaning fewer maintenance tasks can be assigned to each repairperson. Additionally, as the constraint satisfaction probability is relaxed (*i.e.,* *α* increases), expected overtime increases. This represents an important trade-off that decision-makers must consider. To further illustrate the impact of *α* on maintenance assignment, Fig. 12 shows the average maintenance time per repairperson, along with the minimum and maximum values, for varying *α*. It is evident that smaller values of *α* lead to lower average maintenance times per repairperson.

## Conclusions and future research directions

This paper presented a novel mathematical model to jointly optimize fleet maintenance planning, repairperson scheduling, and system-to-mission assignment to minimize the total expected cost incurred. The fleet consists of identical multi-component systems, which undergo maintenance during the break duration to ensure readiness for a set of unique upcoming missions. The resulting decision problem involves: (i) selecting the components within each system to maintain and their corresponding maintenance levels, ensuring reliability requirements are met, (ii) assigning maintenance tasks to repairpersons, and (iii) allocating systems to mission types. The systems include both standard and sensor-monitored components. Standard components follow known lifetime distributions, allowing reliability values to be computed analytically. Sensor-monitored components are continuously tracked by sensors, and a data-driven approach leveraging a deep neural network and Monte Carlo Dropout was used for accurate reliability prediction. To address the complex optimization problem while accounting for uncertainties in maintenance and break durations, a chance-constrained optimization model was proposed. The problem was then reformulated as a large-scale mixed-integer linear program (MILP) using the conditional value-at-risk (CVaR) approximation. Extensive numerical experiments validated the proposed joint model and highlighted the importance of accounting for the inherent uncertainties associated with maintenance and break durations.

This research opens numerous promising avenues for future investigation. The proposed chance-constrained optimization model tends to result in conservative maintenance plans, where repairpersons are likely to complete their assigned maintenance actions well within the break duration. This is particularly true when a high constraint satisfaction probability is required. While this ensures feasibility, it may lead to the hiring of more repairpersons than necessary. To address this, it would be valuable to explore two-stage stochastic optimization models, or other sequential decision methods. However, developing a two-stage optimization model would require the development of efficient solution methods. For extremely difficult and large instances, well-known operations research (OR) techniques such as column generation, branch-and-price schemes, and Benders Decomposition can be used [20, 33]. Finally, the application of various data-driven methods for remaining useful life (RUL) prediction and uncertainty quantification is of great interest. The authors are also exploring domain adaptability, where an RUL predictive model trained in one domain is applied to a system in a different domain using techniques such as transfer learning. This is a crucial research area, as it is not common in many organizations to have a large amount of labeled run-to-failure data.

## Data Availability

The NASA C-MAPSS Jet Engine Simulated Data [44] is an open-access dataset available on the NASA Open Data Portal at the following link: https://data.nasa.gov/Aerospace/CMAPSS-Jet-Engine-Simulated-Data/ff5v-kuh6/about_data. This article’s code is available upon request by contacting the corresponding author.

## Appendix: Data: case study

**Table A.1:** Engine assignment

Components	Test Engine ID	True RUL (cycles)	Predicted RUL (cycles)	$R_{ijk1}$ $(U_{1}=10)$	$R_{ijk2}$ $(U_{2}=25)$	$R_{ijk3}$ $(U_{3}=40)$
$E_{1,1,1}$	17	50	54	1.000	1.000	0.994
$E_{1,1,2}$	18	28	32	1.000	0.966	0.014
$E_{2,1,1}$	20	16	13	0.912	0.000	0.000
$E_{2,1,2}$	21	57	68	1.000	1.000	1.000
$E_{3,1,1}$	24	20	20	1.000	0.054	0.000
$E_{3,1,2}$	27	66	67	1.000	1.000	1.000
$E_{4,1,1}$	31	8	8	0.214	0.000	0.000
$E_{4,1,2}$	32	48	47	1.000	1.000	0.912

**Table A.2:** Component data for standard components

	Initial age $(\varphi _{ijk})$	Initial state $(u_{ijk})$	Weibull shape parameter $(\beta _{ijk})$	Weibull scale parameter $(\eta _{ijk})$
$E_{1,2,1}$	19	1	1.8	30
$E_{1,2,2}$	30	0	1.8	30
$E_{1,2,3}$	17	1	1.8	30
$E_{1,3,1}$	24	0	2.4	40
$E_{1,3,2}$	25	0	2.4	40
$E_{1,4,1}$	42	1	2.2	45
$E_{1,5,1}$	20	0	2.5	30
$E_{2,2,1}$	22	0	1.8	30
$E_{2,2,2}$	20	0	1.8	30
$E_{2,2,3}$	16	1	1.8	30
$E_{2,3,1}$	39	0	2.4	40
$E_{2,3,2}$	24	0	2.4	40
$E_{2,4,1}$	22	1	2.2	45
$E_{2,5,1}$	17	1	2.5	30
$E_{3,2,1}$	21	0	1.8	30
$E_{3,2,2}$	17	1	1.8	30
$E_{3,2,3}$	24	0	1.8	30
$E_{3,3,1}$	26	0	2.4	40
$E_{3,3,2}$	22	1	2.4	40
$E_{3,4,1}$	38	1	2.2	45
$E_{3,5,1}$	18	0	2.5	30
$E_{4,2,1}$	25	0	1.8	30
$E_{4,2,2}$	21	1	1.8	30
$E_{4,2,3}$	24	0	1.8	30
$E_{4,3,1}$	31	0	2.4	40
$E_{4,3,2}$	27	0	2.4	40
$E_{4,4,1}$	43	1	2.2	45
$E_{4,5,1}$	19	1	2.5	30

**Table A.3:** Maintenance duration parameters for different components and maintenance levels

$E_{ijk}$	$t^{p}_{ij2}$		$t^{p}_{ij3}$		$t^{c}_{ij1}$		$t^{c}_{ij2}$		$t^{c}_{ij3}$	
	$\xi _{sc}$	$\xi _{sh}$	$\xi _{sc}$	$\xi _{sh}$	$\xi _{sc}$	$\xi _{sh}$	$\xi _{sc}$	$\xi _{sh}$	$\xi _{sc}$	$\xi _{sh}$
$E_{i1k}$	–	–	41.4	2.56	–	–	–	–	44.4	2.56
$E_{i2k}$	20.5	2.28	31.4	2.78	8.9	2.20	23.5	2.28	34.4	2.78
$E_{i3k}$	18.1	2.17	35.6	2.67	13.0	1.84	21.1	2.17	38.6	2.67
$E_{i4k}$	15.9	1.71	32.1	2.21	14.7	2.29	18.9	2.32	35.1	2.82
$E_{i5k}$	16.7	1.72	33.7	2.22	16.8	1.88	25.7	1.94	37.7	2.44

## Abbreviations

Bi-LSTM: Bi-directional LSTM network
CM: Corrective maintenance
CVaR: Conditional Value-at-Risk
DL: Deep Learning
DNNs: Deep Neural Networks
FSM: Fleet Selective Maintenance
FSMO: FSM Optimization
FSMP: FSM Problem
IM: Imperfect maintenance
LSTM: Long short-term memory network
MCD: Monte Carlo Dropout
MOS: Mission-oriented system
PM: Preventive maintenance
RNN: Recurrent neural network
RUL: Remaining useful lifetime
SAA: Sample Average Approximation
SM: Selective maintenance
SMP: SM problem
